# Nursing and midwifery workforce readiness during a global pandemic: A survey of the experience of one hospital group in the Republic of Ireland

**DOI:** 10.1111/jonm.13461

**Published:** 2021-09-19

**Authors:** Mary Ryder, Paul Gallagher, Barbara Coughlan, Phil Halligan, Suzanne Guerin, Michael Connolly

**Affiliations:** ^1^ School of Nursing, Midwifery and Health Systems University College Dublin Dublin Ireland; ^2^ Department of Nursing St. Vincent's University Hospital Dublin Ireland; ^3^ Ireland East Hospital Group Dublin Ireland; ^4^ School of Psychology University College Dublin Dublin Ireland

**Keywords:** education, leadership, management, mobilization, nursing, survey

## Abstract

**Aim:**

To explore the mobilization of nurses/midwives in a designated hospital group in Ireland during a global pandemic.

**Background:**

The recent global pandemic has resulted in the large‐scale worldwide mobilization of registered nurses and midwives working in the acute care sector. There is a dearth of literature reporting the mobilization of this professional workforce.

**Method:**

Mixed‐methods design using an electronic survey and facilitated discussion across one Irish hospital group.

**Results:**

Eight of 11 hospitals responded to the survey. There was a 2% vacancy rate prior to the pandemic. Mobilization included reconfiguration of clinical areas and redeployment of 9% of the nursing/midwifery workforce within 2 weeks of the pandemic. A total of 11% (*n* = 343) of nurses/midwives were redeployed in 3 months. Nurses/midwives required re‐skilling in infection prevention control, enhancement of critical care skills and documentation.

**Conclusions:**

Three key areas were identified to enable the nursing workforce readiness. These are referred to as the three ‘R's’: Reconfiguration of specific resources, Redeployment of nurses to dedicated specialist areas and Re‐skilling of nurses to safely care for the patients during the pandemic.

**Implications for Nursing Management:**

- A centralized approach to reconfiguration of clinical areas.
- Redeployment is enabled by closing non‐essential departments.
- Hands‐on re‐skilling and reorientating staff are essential.

## 1 INTRODUCTION

The coronavirus (COVID‐19) global pandemic has resulted in the large‐scale worldwide mobilization of Registered Nurses and Midwives working in the acute hospital sector, to meet the urgent needs of patients and families (Jackson et al., 2020). The first phase of the pandemic hit most countries, including Ireland, in Spring 2021. Research to date has examined various countries' response to increasing the health system capacity (Köppen et al., 2021). Health system strategies to expand the health workforce during surges in the pandemic have been compared across countries (Williams et al., 2020). It is widely known that the nursing and midwifery workforce comprises the largest numbers in most health care settings worldwide, the breadth and impact of this action has yet to be established. Adequate workforce or nurse staffing is a match of registered nurse expertise with the needs of the patients being cared for (ANA, 2012). Research studies have outlined an association between higher registered nurse staffing levels in hospitals with better patient outcomes and improved care quality (Griffiths et al., 2016, 2018). However, determining the appropriate nurse staffing requirements has been shown to be weak and major deficits (Griffiths et al., 2018). During Covid 19, nursing management teams were responsible to implement strategies to mobilize the large workforce to meet the unpredictable demands of a pandemic (Wu et al., 2020).

Mobilizing a workforce requires people to take on new roles and be re‐deployed to an unfamiliar area of work, in the provision of direct patient care, with unfamiliar symptomatology, including rapid deterioration and high rates of death. Nurses are required to continually maintain their professional registration requirements, within an environment of uncertainty and fear. The degree of uncertainty related to COVID‐19 resulted in increased difficulty in planning to meet unknown patient nursing needs (Fan et al., 2021). Anecdotal evidence gleaned from members of the research team who hold appointments in the clinical environment, suggests the process for redeployment is met with resistance, enthusiasm, compassion, commitment, anxiety, and understanding from the entire nursing workforce.

## 2 BACKGROUND

Healthcare in the Republic of Ireland is governed by the Department of Health from a policy perspective, operationally the Health Service Executive (HSE), with devolved governance to seven Hospital Groups. One of the seven hospital groups is Ireland East Hospital Group (IEHG). The IEHG, comprises 11 hospitals, with a nursing/midwifery workforce of 4400. The group serves a population of over 1.1 million people. The IEHG acute hospital configuration consists of two model four hospitals, four model three hospitals, two model two hospitals and three specialist hospitals. Table 1 explains the various hospital model configurations (O'Reilly et al., 2015).

**TABLE 1:** Hospital model configurations

Model	Description
Model 1	Model 1 hospitals are community units with subacute inpatient beds that care for patients with rehabilitation, respite, or palliative care needs.
Model 2	This group includes small hospitals that provide inpatient and outpatient care for low risk, differentiated medical patients or are referred on to associated higher complexity facilities.
Model 3	The majority of hospitals in the country are Model 3 general hospitals, admitting 50% of all medical patients. Model 3 hospitals provide emergency department expertise, acute medical, surgical and critical care.
Model 4	There are eight Model 4 hospitals that function as tertiary referral centres in Ireland. They provide emergency department expertise, acute medical, surgical and critical care plus specialist and supra‐regional care. A considerable volume of their patient workload remains inpatient admissions for routine specialist inpatient care.

While there has been plentiful literature related to nursing during the COVID‐19 pandemic, there is a dearth of literature reporting nursing management roles to implement the strategy for workforce mobilization. That said, a recent study by Wu et al. (2020), in China, described the nursing management strategy to transform a hospital to a COVID designated site, which included the reconfiguration of clinical areas, creating a supply of nursing staff and preparing training for nurses to meet clinical demands of their roles. What is worth noting is that the limited literature that previously examined the mobilization of healthcare staff during the swine flu (H1N1) influenza crisis (Considine et al., 2011), and the severe acute respiratory syndrome (SARS) crisis (Fitzgerald et al., 2012) confirms that lessons have not been learned and there is an evident lack of research in this area.

A major emergency management plan is an approach used by organisations to ensure appropriate planning, preparedness, capacity, training and coordination are in place to enable it to meet any challenges posed (Health Service Executive, 2020). No current framework exists to support decisions related to the redeployment of staff, particularly for medium to long‐term purposes. This is despite nurses and midwives in an organisation having a contractual obligation to accept redeployment in emergency situations. The current practice is for senior nursing and midwifery management to identify staff for redeployment to other areas of the organisation to deliver patient care. It is critical to address the gap in understanding the impact of redeployment on nurses to gain greater knowledge for workforce planning for potential future pandemics.

## 3 AIM

The aim of the research was to explore nursing workforce readiness (preparedness) in one hospital group in Ireland during a global pandemic.

The objectives of the study were as follows:

- To quantify staffing and mobilization of nurses/midwives across a large hospital group in the Republic of Ireland during the global pandemic
- To identify the extent of the reconfiguration of services across the hospital group
- To identify education priorities and resources provided for nursing/midwifery staff

## 4 METHODS

This study used a mixed methods design. A specifically designed questionnaire was used for this study. Drawing from the literature and professional expertise the 20‐item “Nursing Workforce Readiness Survey” was created by the research team, some of whom are senior nurse managers, using Qualtrics® software (Supporting Information S1). The survey was tested for face validity among an additional three Directors of Nursing. Reliability was checked at analysis. A second phase of the study included an additional reflective component, where the results from the questionnaire were presented to the Directors of Nursing (DON) and Midwifery (DOM), whose comments were recorded and included in reporting of the results.

A convenience sample of the DON and DOM in the hospital group was selected. A link to the cloud‐based survey was distributed to the 11 Directors of Nursing/Midwifery across the hospital group during September to November 2020. The survey was circulated electronically, with an exhaustive sampling approach adopted and all possible participants in the hospital group invited to take part.

Qualitative data were recorded from participants at a scheduled meeting following a presentation of the survey responses. All eight participants were present.

### 4.1 Ethical considerations

A review of a declaration of exemption from full ethical review was accepted by the University Research Ethics Committee, LS‐E‐20‐84‐Ryder. Participation information was provided at the beginning of the survey. Participants were informed that completion of the survey implied consent.

An additional declaration of exemption from full ethical review was accepted by the University Research Ethics Committee to collate anonymous feedback from the participants following the presentation of the results. Participants were advised in advance of the feedback presentation that their responses were recorded and were requested to state that they did not wish for their responses to contribute to the research during the discussion.

### 4.2 Data analysis

Quantitative data were downloaded from Qualtrics, and analysis was conducted using the software package IBM SPSS®, Statistics Version 24. Data were checked and cleaned for analysis. Four blank survey responses were removed. Descriptive statistics were used to describe, compare and summarize participant responses.

Qualitative data were recorded, transcribed and analysed by two members of the research team. Data were analysed using Braun and Clarke (2006) thematic analysis framework.

## 5 RESULTS

Eight of 11 (73%) hospitals responded to the survey (Table 2). The majority (*n* = 5; 63%) of responses were from model three hospitals.

**TABLE 2:** Hospital configurations

Hospital model	Frequency
2	1
3	5
4	1
Maternity services	1
Total	8

### 5.1 Nursing vacancies

In order to understand the context in which the Health Care Group organised and managed its Nursing and Midwifery workforce, the participant hospitals were requested to provide the pre‐COVID‐19 staffing configuration as identified in Table 3. Participants were also requested to identify pre‐COVID nursing vacancies using the same configuration. There were a total of 83 nursing vacancies in participating hospitals prior to COVID‐19.

**TABLE 3:** The pre‐COVID staffing configuration across hospitals were as follows

Pre‐COVID number whole time equivalent (WTE)	Hospital model				
	2	3	4	Maternity services	Total
	M (n)	M (n)	M (n)	M (n)	M (n)
RGN/RM	116 (116)	239 (1196)	625 (625)	251 (251)	273 (2187)
CNM/CMM 1	11 (11)	17 (84)	55 (55)	44 (44)	24 (195)
CNM/CMM 2	15 (15)	29 (145)	82 (82)	62	38 (305)
CNM/CMM 3	0 (0)	5 (24)	8	8	5 (40)
CNS/CMS	7 (7)	13 (66)	63	10	18 (144)
cANP/RANP or cAMP/RAMP	4 (4)	6 (30)	21 (21)	6 (6)	8 (61)
ADON/ADOM	5 (5)	6 (32)	11 (11)	9 (9)	7 (57)
Other	2 (2)	4 (18)	1 (1)	1 (1)	4 (36)
Nurses (all grades) in ICU or equivalent	10 (9)	19 (77)	105 (105)	17 (17)	30 (209)
Nurses (all grades) in ED	14 (14)	28 (140)	90 (90)	17 (17)	33 (260)

Abbreviations: ADON/ADOM, assistant director of nursing/assistant director of midwifery (ADOM); cAMP/RAMP, candidate advanced midwife practitioner/registered advanced midwife practitioner; cANP/RANP, candidate advanced nurse practitioner/registered advanced nurse practitioner; CNM/CMM, clinical nurse manager/clinical midwife manager; CNS/CMS, clinical nurse specialist/clinical midwife specialist; ED, emergency department; ICU, intensive care unit; RGN/RM, registered general nurse/registered midwife.

### 5.2 Corporate planning

When questioned whether or not their organisation had a documented contingency plan to escalate intensive care units (ICU) in the event of a pandemic half (*n* = 4; 50%) of all the hospitals reported that they had this prior to March 2020. Seventy‐five percent (*n* = 6) of organisations reported adopting a collaborative decision‐making approach to decide on the reconfiguration of clinical areas (Figure 1). The majority (*n* = 6; 75%) of organisations indicated that both ward design, and clinical skills of the nurses working in the ward areas, informed decisions related to the reconfiguration of clinical areas as opposed to a surge in activity.

### 5.3 Redeployment/mobilization of nurses

Participants were requested to list the areas where staff were mobilized from. Responses indicated that hospitals mobilized staff from within their own organisation to critical areas including emergency departments, critical care and newly designated COVID‐19 areas (Table 4). Responses identified that staff were redeployed from a variety of clinical areas that were closed including outpatient departments, operating theatres, endoscopy services and nurse education and practice development departments. Clinical Nurse Specialists (CNS) were also redeployed from specialist services that were closed for outpatient visits. Two hospitals employed a number of agency nurses to increase the numbers of nurses working in critical areas.

**TABLE 4:** Immediate nursing mobilization to target areas from 1 March to 15 March 2020

Immediate staffing redeployment to increase capacity in departments	Hospital model				
	2	3	4	Maternity services	Total
	M (n)	M (n)	M (n)	M (n)	M (n)
Emergency department	0 (0)	2 (10)	30 (30)	0 (0)	6 (40)
ICU/HDU	0 (0)	7 (36)	10 (10)	0 (0)	6 (46)
Other clinical areas	0 (0)	23 (116)	81 (81)	4 (4)	29 (201)

Abbreviations: HDU, high dependency unit; ICU, intensive care unit (ICU).

The total number of nurses/midwives working across the eight hospitals in the study was reported as 3019, with 83 vacancies. Within the first 2 weeks of the pandemic, 287 (9%) members of the nursing/midwifery workforce were redeployed to alternative working areas. Overall, 11% (*n* = 343) of the nursing/midwifery workforce were redeployed across the eight hospitals (Table 5).

**TABLE 5:** Total number of nurses mobilized in hospitals between 1 March and 1 June 2020

Total staffing redeployment to increase capacity in departments	Hospital model				
	2	3	4	Maternity services	Total
	M (n)	M (n)	M (n)	M (n)	M (n)
All clinical areas	33 (33)	32 (162)	130 (130)	018 (18)	43 (343)

*Note*: Total number of staff that were redeployed from 1 March to 1 June 2020.

As part of the survey, the hospitals were asked to identify three education priorities for nursing staff at the onset of the pandemic. The first priority identified by all organisations was related to infection prevention control, including procedures for “don and doff” of personal protective equipment. This second education priority was the provision of instruction to enhance nursing staff in critical care skills including care of the patient receiving invasive and non‐invasive ventilation. The third priority was to upskill Registered Nurses who were redeployed from other clinical areas, who were recent appointments to the hospital, many from the non‐acute care sector, particularly from nursing homes.

Participants were requested to identify the education resources in place for redeployed staff and explain the mode of delivery of the education available. Responses indicated that the education was provided by Centres for Nurse Education, Nurse Practice Development from both local and regional areas, and higher education institutions. Two hospitals identified the use of online educational resources to support upskilling of staff.

### 5.4 Feedback from participants

The results of the survey were presented to the group of participants at a scheduled meeting. There were two key discussion topics raised by the group, namely redeployment and education.

#### 5.4.1 Redeployment

The participant group (*n* = 8) expressed surprise at what they referred to as the small percentage of staff redeployed.

I cannot believe it's only 9–11%, we phoned every available CNS (DOM 3)

They had anticipated that this number would be approximately 25% of staff. The rationale for anticipating a higher percentage was explained by the recollection of identifying and calling every possible CNS, they however noted that this group comprised 5% of all nurses/midwives.

A number of participants (*n* = 6) discussed that perhaps the survey did not capture what was described as “hidden redeployment.” This was described as situations where out‐patient based nurses/midwives were redeployed to ward areas to enable ward‐based nurses to be redeployed to other wards with a higher patient acuity such as non‐invasive ventilation. As one participant stated:

I think it misses the double redeployment element where we put CNS into stable wards to move the ward staff to the more complex wards, there was a hidden element to redeployment if you like. (DON 6)

One participant stated, “it wasn't all about critical care.” This comment was met with a lot of agreement. When nurses were moved between wards, this was not identified as redeployment in the survey; however, upon reflection, it was described as “double redeployment.”

One participant reflected that there were a number of discussions prior to the pandemic where concerns were raised about critical care staff shortages. The same participant noted that despite the concerns, the group were able to mobilize staff to critical care during a crisis. The group agreed with this commentary.

#### 5.4.2 Education and training

The need for education resources was highlighted among the group, particularly in clinical educator roles where the availability of expertise to provide hands on skills training was needed. It was discussed that practical “hands‐on” focused skills training was the primary requirement across the organisations.

A consensus among the group was related to the importance of the Clinical Facilitator roles and availability. This was particularly expressed amongst DON in model three hospitals as these were the education resources needed locally to prepare staff.

Not having clinical facilitators was a real problem for us, we struggled to find someone to teach clinical skills (DON 4)

It was agreed that there is a lack of funding for these positions and the group expressed the need for permanent positions to be actioned.

## 6 DISCUSSION

This study explored the nurses and midwives experience of mobilization in the first wave (1 March 2020 to 1 August 2020) of a global coronavirus pandemic in a large hospital group in the Republic of Ireland. Three key deliverables were required to occur almost simultaneously to enable mobilization of the nursing and midwifery workforce. They were Reconfiguration, Redeployment and Re‐skilling. The clinical areas required reconfiguration from their previous specialist derogation to dedicated COVID‐19 suitable clinical environments. Overall, during the COVID‐19 pandemic, nurses and midwives' managers reported the urgent need to mobilize staff from other areas within their hospitals to their intensive care departments/units to meet the huge demand for intensive care nursing. In addition, they reported a crucial need to re‐skill nurses and midwives in specific infection control skills including donning and doffing of personal protective equipment (PPE), educating and re‐skilling nurses/midwives for critical care areas and new documentation.

### 6.1 Reconfiguration

This research identified that a collaborative decision‐making approach was applied to reconfigure clinical wards to accommodate a predictive surge in critical care and emergency departments as the COVID‐19 virus increased in the community. The factors influencing ward reconfiguration was the ward design and the clinical skills of nurses. Hospital resource planning is complex at the best of times, but in the midst of a disaster has the potential to increase the loss of lives due to unavailability of specific resources and or skills (Aghapour et al., 2019). Arabi et al. (2021) argue that the best approach to management of critical care surges is to prevent them by implementing a centralized approach to management of admission to critical care. In contrast, Hattke and Martin (2020) argue whether a centralized or a decentralized approach is most appropriate is a matter of much debate. It is known that some form of coordination, collaboration or cooperation is necessary during a crisis (Kapucu et al., 2010; Martin et al., 2016) but the degree to which these occurred to bring about collective action within one hospital group is currently unknown. The literature would therefore attest to the centralized collaborative approach applied by the hospitals in the reconfiguration of clinical areas.

The ongoing shortage of nursing and midwives has attracted the attention of the Organisation for Economic Co‐operation and Development (OECD), which projects a significant worldwide nursing shortage by 2030, with Ireland, having a projected nurse and midwife shortage of 9.1% (Scheffler & Arnold, 2019). The existing shortage of nurses had already provided a massive strategic risk to the effective functioning of the healthcare system, with many hospitals already facing a staffing crisis, when the COVID‐19 pandemic hit (Jilani, 2019). However, the results of this study highlight that within one hospital group prior to COVID‐19, there were only 83 (2%) whole time equivalent (WTE) nursing positions vacant prior to the pandemic.

### 6.2 Redeployment

This is the first study to quantify the level of mobilization of nurses and midwives during the pandemic. The redeployment of employees was one of the core elements of the response to COVID‐19 and reflects the leadership of the nursing executive teams in participating organisations. The findings identified that 9% of the workforce were mobilized within the first 2 weeks. The nursing and midwifery staff were mobilized internally to designated COVID‐19 specialist areas. This process resonates with Minissian et al. (2020) whereby redeployment efforts took centre stage for optimizing staffing needs and the surge planning and redeployment efforts led by senior leaders were imperative to ensure crucial staffing needs were achieved.

At a national level, non‐essential services (e.g., outpatient clinics and elective surgery) were cancelled or postponed. Arabi et al. (2021) recommend this systematic centralized approach to enable the health system “flex” to accommodate increased demand for hospitalized care. This was reflected in the results of this research where participants indicated that mobilization to COVID‐19 designated areas was accommodated through internal redeployment of nurses and midwives whose positions were curtailed or temporarily suspended. The literature has reported consistent findings related to the nursing workforce mobilization. Retzlaff (2020) also noted the need to redeploy perioperative team members to other units and remarked that a common thread at health care facilities across the country was the willingness of staff members to pitch in and do what was necessary to help their communities respond to the COVID‐19 pandemic, whether it was temporarily transitioning to a different unit, helping their colleagues with patient positioning, and donning or doffing PPE. To increase the number of nursing staff, nursing interns and retired nurses were used to fill vacancies (Propper et al., 2020). This was not consistent with the experience reported from participants in this research who identified that mobilization was accommodated through internal redeployment.

In the current study, the impact of nurse staffing levels on patient outcomes is unknown in the hospital group, given the scale, speed and age profile most affected during the first wave of the pandemic. Of note, there were 8582 deaths registered in Ireland during this period (Quarter 2, 2020) and of these 1227 deaths were assigned an Underlying Cause of Death (UCOD) of COVID‐19, an increase of 14.1% (or 1063 deaths) from Quarter 2 2019 was also reported (Central Statistics Office, 2020).

### 6.3 Re‐skilling

The need to address related training and education in this study were infection control, intensive care nursing and orientation for nurses redeployed or nurses returning to professional practice relates to the challenges in providing support to reduce the gaps in critical knowledge by Chen et al. (2020). Danielis et al. (2021) described the experience of Italian nurses' redeployment who felt unsupported due to the absence of education and training in skills and documentation required to work in their new clinical area. This research supports the literature identifying that training and up‐skilling of nurses was a feature of the measures taken by the profession during the pandemic. This research identified three key skill enhancement areas prioritized, infection and prevention and control, critical care skill enhancement and documentation updates.

The virus, COVID‐19, was identified as a highly Infectious disease (WHO, 2020). While most nurses and midwives are familiar with a variety of infectious disease PPE related procedures, COVID‐19 was highly contagious, therefore, required focused skills related to donning and doffing full PPE.

This research adds to the existing literature by identifying the specific requirement for “hands‐on” skills training in lower acuity organisations. The participating nurse leaders expressed explicitly that skills requirements were not all critical care focused. In contrast, Jackson et al. (2020) identified that it was crucial to have adequate learning resources available to support staff who were redeployed to new areas in particular intensive care. Patient safety no matter what the circumstances are is paramount.

Participants in this study expressed a preference for hands‐on skills training to re‐skill redeployed staff. Only two organisations availed of the on online education made available. Almomani et al. (2021) reported that the upskilling of non‐critical care nurses was conducted using simulation‐based education and consisted of completing a mandatory online critical care awareness module. Similarly, Danielis et al. (2021) reported that nurses were required to gain competence individually as the only resource available was distance education. The findings in this research identified that education and skills acquisition was supported by clinical educators and academic partners. Participants highlighted a need for investment in specific clinical teaching roles to enable essential upskilling of staff.

### 6.4 Limitations

The findings emerging from this study need to be viewed within the context of its limitations, namely that this study reports on one period of time at the start of the COVID‐19 pandemic and during what is now considered the first wave of the pandemic. In Ireland there have been two other substantial waves of COVID‐19 which have impacted greatly on the provision of health care within the IEHG. Furthermore, the data presented in the paper represents eight of the 11 hospitals in one group, and the data gathered relating to one of the maternity care services was limited to protect anonymity. Future research will need to consider Nursing and Midwifery services for all Hospital Groups in the Republic of Ireland during the past 12 months and examine the experiences of the Nurses and Midwives who were redeployed during the pandemic to gain a greater understanding for future service development.

## 7 CONCLUSIONS

Nurses are the backbone of healthcare delivery and demonstrated willingness and flexibility in adapting to new ways of working during the first wave of COVID‐19 pandemic. This study revealed that workforce readiness during the first wave of a global pandemic was influenced by many factors, for example, a documented contingency plan, collaborative decision making, ward design, and the upskilling of nurses' clinical skill set. On reflection many of the Directors of Nursing were surprised at the low number of nurses redeployed to different departments but it was suggested that there may be some hidden redeployment as some nurses adopted different roles within their usual work environments. Educators, more importantly hands‐on clinical educators were identified as essential to the successful mobilization of nursing staff across the organisation. Nursing and midwifery staff readiness to cooperate in this crisis is testament to their commitment to assist patients, families and colleagues to respond to COVID‐19 tsunami that gripped the nation at the time.

### 7.1 Implications for nursing management

This research identified three key factors for consideration by nursing management to mobilize the nursing workforce in response to a pandemic. Reconfiguration of clinical areas to respond to a surge in hospital capacity is a collaborative approach at management level. Closure of non‐emergency services in the organisation results in availability of staff for redeployment. Hands‐on skills training and refreshment of updated documentation is essential to support staff being redeployed to clinical areas. Organisational measures at macro and micro levels need to be considered if nursing and midwifery are to be adequately prepared for future surges or pandemics.

## CONFLICT OF INTEREST

The authors wish to declare that there are no conflicts of interest.

## ETHICAL APPROVAL

A declaration from full ethical review was accepted by the Office of Research Ethics at the Higher Education Institution, reference LS‐E‐20‐84‐Ryder.

## Supporting information


**Data S1.** Supporting Information

## Data Availability

The data that support the findings of this study are available on request from the corresponding author. The data are not publicly available due to privacy or ethical restrictions.

## References

[jonm13461-bib-0001] Aghapour, A. H. , Yazdani, M. , Jolai, F. , & Mojtahedi, M. (2019). Capacity planning and reconfiguration for disaster‐resilient health infrastructure. Journal of Building Engineering, 26, 100853. 10.1016/j.jobe.2019.100853

[jonm13461-bib-0033] Almomani, E. , Sullivan, J. , Hajjieh, M. , & Leighton, K. (2021). Simulation‐based education programme for upskilling non‐critical care nurses for COVID‐19 deployment. BMJ Simulation & Technology Enhanced Learning, 7(5), 319–322. 10.1136/bmjstel-2020-000711 PMC8936668 35515720

[jonm13461-bib-0002] ANA . (2012). Principles for nurse staffing (2nd ed.). American Nurses Association. https://cdn2.hubspot.net/hubfs/4850206/PNS3E_ePDF.pdf

[jonm13461-bib-0003] Arabi, Y. M. , Azoulay, E. , Al‐Dorzi, H. M. , Phua, J. , Salluh, J. , Binnie, A. , Hodgson, C. , Angus, D. C. , Cecconi, M. , Du, B. , Fowler, R. , Gomersall, C. D. , Horby, P. , Juffermans, N. P. , Kesecioglu, J. , Kleinpell, R. M. , Machado, F. R. , Martin, G. S. , Meyfroidt, G. , … Citerio, G. (2021). How the COVID‐19 pandemic will change the future of critical care. Intensive Care Medicine, 47(3), 282–291. 10.1007/s00134-021-06352-y 33616696 PMC7898492

[jonm13461-bib-0004] Braun, V. , & Clarke, V. (2006). Using thematic analysis in psychology. Qualitative Research in Psychology, 3(2), 77–101. 10.1191/1478088706qp063oa

[jonm13461-bib-0037] Central Statistics Office (CSO). (2020). Impact of COVID‐19 on Vital Statistics Quarter 2 2020. Registration of Deaths in Ireland. https://www.cso.ie/en/releasesandpublications/in/vs/informationnote-impactofcovid-19onvitalstatisticsquarter22020/

[jonm13461-bib-0005] Chen, S.‐C. , Lai, Y.‐H. , & Tsay, S.‐L. (2020). Nursing perspectives on the impacts of COVID‐19. Journal of Nursing Research, 28(3), e85. 10.1097/NRJ.0000000000000389 32398577

[jonm13461-bib-0006] Considine, J. , Shaban, R. Z. , Patrick, J. , Holzhauser, K. , Aitken, P. , Clark, M. , Fielding, E. , & FitzGerald, G. (2011 Oct). Pandemic (H1N1) 2009 influenza in Australia: Absenteeism and redeployment of emergency medicine and nursing staff. Emergency Medicine Australasia, 23(5), 615–623. 10.1111/j.1742-6723.2011.01461.x 21995477

[jonm13461-bib-0007] Danielis, M. , Peressoni, L. , Piani, T. , Colaetta, T. , Mesaglio, M. , Mattiussi, E. , & Palese, A. (2021). Nurses' experiences of being recruited and transferred to a new sub‐intensive care unit devoted to COVID‐19 patients. Journal of Nursing Management, 29, 1149–1158. 10.1111/jonm.13253 33480143 PMC8013465

[jonm13461-bib-0009] Fan, E. M. P. , Nguyen, N. H. L. , Ang, S. Y. , Aloweni, F. , Goh, H. Q. I. , Quek, L. T. , Ayre, T. C. , Pourghaderi, A. R. , Lam, S. W. , & Ong, E. H. M. (2021). Impact of COVID‐19 on acute isolation bed capacity and nursing workforce requirements: A retrospective review. Journal of Nursing Management, 29, 1220–1227. 10.1111/jonm.13260 33480121 PMC8013355

[jonm13461-bib-0036] FitzGerald, G. , Aitken, P. , Shaban, R. Z. , Patrick, J. , Arbon, P. , McCarthy, S. , Clark, M. , Considine, J. , Finucane, J. , Holzhauser, K. , & Fielding, E. (2012). Pandemic (H1N1) influenza 2009 and australian emergency departments: Implications for policy, practice and pandemic preparedness. Emergency Medicine Australasia, 24(2), 159–165. 10.1111/j.1742-6723.2011.01519.x 22487665

[jonm13461-bib-0010] Griffiths, P. , Ball, J. , Bloor, K. , Böhning, D. , Briggs, J. , Dall'Ora, C. , Iongh, A. D. , Jones, J. , Kovacs, C. , Maruotti, A. , Meredith, P. , Prytherch, D. , Saucedo, A. R. , Redfern, O. , Schmidt, P. , Sinden, N. , & Smith, G. (2018). Nurse staffing levels, missed vital signs observations and mortality in hospital wards: Retrospective longitudinal observational study using routinely collected data. Health Service Delivery Research Journal, 6(38).

[jonm13461-bib-0011] Griffiths, P. , Ball, J. , Drennan, J. , Dall'Ora, C. , Jones, J. , Maruotti, A. , Pope, C. , Recio Saucedo, A. , & Simon, M. (2016). Nurse staffing and patient outcomes: Strengths and limitations of the evidence to inform policy and practice. A review and discussion paper based on evidence reviewed for the National Institute for health and care excellence safe staffing guideline development. International Journal of Nursing Studies, 63, 213–225. 27130150 10.1016/j.ijnurstu.2016.03.012

[jonm13461-bib-0013] Hattke, F. , & Martin, H. (2020). Collective action during the Covid‐19 pandemic: The case of Germany's fragmented authority. Administrative Theory & Praxis, 42, 4614–4632. 10.1080/10841806.2020.1805273

[jonm13461-bib-0035] Health Service Executive. (2020). Hospitals Major Emergency Plan. Retrieved from https://www.hse.ie/eng/services/list/3/emergencymanangement/hosp

[jonm13461-bib-0015] Jackson, D. , Bradbury‐Jones, C. , Baptiste, D. , Gelling, L. , Morin, K. , Neville, S. , & Smith, G. D. (2020). Life in the pandemic: Some reflections on nursing in the context of COVID‐19. Journal of Clinical Nursing, 29, 2041–2043. 10.1111/jocn.15257 32281185 PMC7228254

[jonm13461-bib-0016] Jilani, S. (2019). The management and administrative practices resulting in the exit of nurses from the Irish healthcare system—A review. DBS Business Review, 3, 17–29. 10.22375/dbr.v3i0.57 https://dbsbusinessreview.ie/index.php/journal/article/view/57

[jonm13461-bib-0017] Kapucu, N. , Arslan, T. , & Demiroz, F. (2010). Collaborative emergency management and national emergency management network. Disaster Prevention and Management: An International Journal, 19(4), 452–468. 10.1108/09653561011070376

[jonm13461-bib-0018] Köppen, J. , Hartl, K. , & Maier, C. B. (2021). Health workforce response to Covid‐19: What pandemic preparedness planning and action at the federal and state levels in Germany? The International Journal of Health Planning and Management, 36(S1), 71–91. 10.1002/hpm.3146 PMC8250947 33735509

[jonm13461-bib-0019] Martin, E. , Nolte, I. , & Vitolo, E. (2016). The four cs of disaster partnering: Communication, cooperation, coordination and collaboration. Disasters, 40(4), 621–643. 10.1111/disa.12173 26749291

[jonm13461-bib-0020] Minissian, M. B. , Ballard‐Hernandez, J. , Coleman, B. , Chavez, J. , Sheffield, L. , Joung, S. , Parker, A. , Stepien, S. J. , Romero, J. , Floríndez, L. I. , & Simons, C. D. (2020). Multispecialty nursing during COVID‐19: Lessons learned in Southern California. Nurse Leader, 19, 170–178. 10.1016/j.mnl.2020.08.013 32922217 PMC7476460

[jonm13461-bib-0021] O'Reilly, O. , Cianci, F. , Casey, A. , Croke, E. , Conroy, C. , Keown, A.‐M. , Leane, G. , Kearns, B. , O'Neill, S. , & Courtney, G. (2015). National acute medicine programme—Improving the care of all medical patients in Ireland. Journal of Hospital Medicine, 10, 794–798. 10.1002/jhm.2443 26271470

[jonm13461-bib-0034] Propper, C. , Stoye, G. , & Zaranko, B. (2020). The wider impacts of the coronavirus pandemic on the NHS. Fiscal Studies, 41(2), 345–356. 10.1111/1475-5890.12227 32836538 PMC7301040

[jonm13461-bib-0038] Retzlaff, K. J. (2020). Staffing and orientation during the COVID‐19 pandemic. AORN Journal, 112(3), 206–211. 10.1002/aorn.13148 32857410 PMC7460928

[jonm13461-bib-0024] Scheffler, R. , & Arnold, D. (2019). Projecting shortages and surpluses of doctors and nurses in the OECD: What looms ahead. Health Economics, Policy, and Law, 14(2), 274–290. 10.1017/S174413311700055X 29357954

[jonm13461-bib-0029] Williams, G. A. , Maier, C. B. , Scarpetti, G. , de Belvis, A. G. , Fattore, G. , Morsella, A. , Pastorino, G. , Poscia, A. , Ricciardi, W. , & Silenzi, A. (2020). What strategies are countries using to expand health workforce surge capacity during the Covid19 pandemic. Eurohealth, 26(2), 5157. https://apps.who.int/iris/bitstream/handle/10665/336263/Eurohealth2622020eng.pdf#page=53

[jonm13461-bib-0030] World Health Organization (WHO) . (2020). State of the world's nursing 2020: investing in education, jobs and leadership. World Health Organization, https://www.who.int/publications/i/item/9789240003279

[jonm13461-bib-0031] Wu, X. , Zheng, S. , Huang, J. , Zheng, Z. , Xu, M. , & Zhou, Y. (2020). Contingency nursing management in designated hospitals during COVID‐19 outbreak. Annals of Global Health, 86(1), 1–5. 10.5334/aogh.2918 32676299 PMC7333556

