# Supplementary material for: Nursing and midwifery workforce readiness during a global pandemic: A survey of the experience of one hospital group in the Republic of Ireland
Source: J Nurs Manag. 2021 Sep 19;30(1):25–32. doi: 10.1111/jonm.13461 (PMC8646494; doi:10.1111/jonm.13461)
Supplement: Supplementary file 1 — Data S1. Supporting Information [file JONM-30-25-s001.docx]

NURSING WORKFORCE READINESS IN IRELAND EAST HOSPITAL GROUP SURVEY

Q1 Hospital Model (Please select one option)

- 2
- 3
- 4
- Other (Please specify) ________________________________________________

Start of Block: STAFFING

Q2 Pre-COVID number Whole Time Equivalent (WTE) Nurses/Midwives employed (please enter number in box below each selection)

- a. RGN/RM (Staff Nurse/Midwife all grades) _________________________
- b. CNM/CMM1 ________________________________________________
- c. CNM/CMM 2 (or equivalent) ___________________________________
- d. CNM/CMM 3 ________________________________________________
- e. CNS/CMS (or CNM/CMM working in CNS/CMS position) ______________
- f. cANP/RANP or cAMP/RAMP ____________________________________
- g. ADON/ADOM ________________________________________________
- h. other ________________________________________________

Q3 Pre-COVID WTE number of Nursing/Midwifery permanent vacancies">(please enter number in box below each selection)

- a. RN/RM (Staff Nurse/Midwife all grades) ___________________________
- b. CNM/CMM1 ________________________________________________
- c. CNM/CMM 2 ________________________________________________
- d. CNM/CMM 3 ________________________________________________
- e. CNS/CMS (or CNM/CMM working in CNS/CMS position) _____________
- f. cANP/RANP or cAMP/RAMP ____________________________________
- g. Other ________________________________________________

Q4 Pre-COVID WTE number of Nurses working in ICU or equivalent (all grades)

________________________________________________________________

________________________________________________________________

Q5 Pre-COVID WTE number of permanent Nurse vacancies in ICU or equivalent (all grades)

________________________________________________________________

________________________________________________________________

Q6 Pre-COVID WTE number of Nurses (all grades) working in the Emergency Department (ED)

________________________________________________________________

________________________________________________________________

Q7 Pre-COVID WTE number of permanent Nurse vacancies in ED (all grades)

________________________________________________________________

________________________________________________________________

End of Block: STAFFING

Start of Block: SERVICES AND CONFIGURATION

Q8 >Decisions to reconfigure* clinical areas was made by (*designating wards/units to specifically care for patients suspected or confirmed with COVID 19)

- a. Hospital Executive Management Team
- b. Nursing/Midwifery Executive Team/Nursing/Midwifery Management Team
- c. Both
- d. Other (please specify) ________________________________________________

Q9 Did you have a documented contingency plan to escalate ICU capacity in the event of a Pandemic prior to March 2020?

- Yes
- No

Q10 Can you outline the immediate (1st-15th March 2020) staffing redeployment that occurred in order to increase Emergency Department capacity

[please indicate a) the number of staff increased in the department b) please indicate where the staff were redeployed from eg. CNS, Education, another clinical area]

- a) Increase in number by ________________________________________________
- b) Redeployment from what areas (please list all that apply) ________________________________________________

Q11 Can you outline the immediate (1st-15th March 2020) staffing redeployment that occurred in order to increase ICU/HDU capacity?

[please indicate a) the number of staff increased in the department b) please indicate where the staff were redeployed from eg. CNS, Education, another clinical area]

- a) Increase in number by ________________________________________________
- b) Redeployment from what areas (please list all that apply) ________________________________________________

Q12 Can you outline the immediate (1st-15th March 2020) staffing redeployment that occurred in order to increase the capacity in other clinical areas?

[please indicate a) the number of staff increased in the department b) please indicate where the staff were redeployed from eg. CNS, Education, another clinical area]

- a) Increase number by ________________________________________________
- b) Redeployment from what areas (please list all that apply) ________________________________________________

Q13 What considerations were taken that informed your immediate decision to reconfigure the clinical areas? (Please select one option)

- a) Ward design (for infection control)
- b) Staff clinical skills
- c) Both ward design and staff clinical skills
- d) Surge in activity
- e) Other (please specify) ________________________________________________

Q14 As of 1st June 2020, what was the WTE number of Nurses working in ICU/HDU (or equivalent)

________________________________________________________________

________________________________________________________________

Q15 As of 1st June 2020, what was the WTE number of Nurses working in Emergency Department?

________________________________________________________________

________________________________________________________________

Q16 As of 1st June 2020, what was the Whole Time Equivalent (WTE) number of Nurses/Midwives (all grades) employed?

(please note we are happy to accept an actual number or an estimated number)

- Actual number ________________________________________________
- Estimated Number ________________________________________________

Q17 How many nurses/midwives (total WTE) have been redeployed in your organisation during the period of 1st March to 1st June 2020?

(please note we are happy to accept an actual number or an estimated number)

- Actual number ________________________________________________
- Estimated number ________________________________________________

End of Block: SERVICES AND CONFIGURATION

Start of Block: EDUCATION

Q18 Can you please list the first three education priorities identified to support nurse/midwife redeployment needs?

- 1. First Priority ________________________________________________
- 2. Second Priority ________________________________________________
- 3. Third priority ________________________________________________

Q19 Can you please list the education resources that were in place for nurses/midwives being redeployed on 1st June 2020?

________________________________________________________________

________________________________________________________________

Q20 Can you briefly describe the mode of delivery of education

(e.g. face to face, on-line) available for nurses/midwives in your organisation?

________________________________________________________________

________________________________________________________________

End of Block: EDUCATION
